# Janus Ligand-Tethered Nanoparticles at Liquid–Liquid Interfaces

**DOI:** 10.1021/acs.jpcb.3c01943

**Published:** 2023-05-29

**Authors:** Małgorzata Borówko, Tomasz Staszewski, Joanna Tomasik

**Affiliations:** Department of Theoretical Chemistry, Institute of Chemical Sciences, Faculty of Chemistry, Maria Curie-Skłodowska University, 20-031 Lublin, Poland

## Abstract

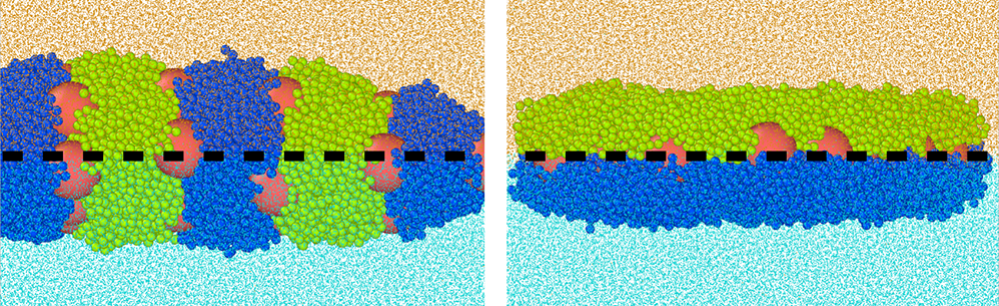

We investigate the structural properties of Janus ligand-tethered nanoparticles at liquid–liquid interfaces using coarse-grained molecular dynamics simulations. The effect of interactions between different chains and liquids is discussed. We consider the Janus particles with symmetrical interactions with the liquids which correspond to supplementary wettability and particles with uncorrelated interactions. Simulation results indicate that the Janus hairy particles trapped in the interface region have different configurations characterized by the vertical displacement distance, the orientation of the Janus line relative to the interface, and the particle shape. The Janus hairy particles present abundant morphologies, including dumbbell-like and typical core–shell, at the interface. The shape of adsorbed particles is analyzed in detail. The simulation data are compared with those predicted by a simple phenomenological approach. This work can promote the applications of Janus hairy particles in nanotechnology.

## 1 Introduction

Polymer-tethered (hairy) nanoparticles have attracted considerable scientific and technological interest in the past few decades.^1,2^ These particles are composed of a core and a layer of polymer chains grafted on the core surface. These hybrid structures naturally combine the unique features of both polymers and cores. Due to their special properties, hairy particles have numerous technological applications, such as the stabilization of emulsions, production of composites, sensors, drug delivery systems, pollution removal, etc.^1,2^ Effectively controlling these processes requires understanding the behavior of hairy particles in different systems. From a practical point of view, it is very important to study the particles at fluid–fluid interfaces.^3−9^ Biological and medical applications of hairy particles require an understanding of their behavior at interfaces and the transfer to cells.^2^ On the other hand, the particle-laden layers can be used to solve some basic problems in condensed matter physics. For example, the confinement of the particles in a quasi-two-dimensional interface between two fluids causes the emergence of completely new behaviors and properties that are not observed in the three-dimensional systems.^8^ Progress in the synthesis methodology enables the production of various particles that differ in shape, size, or surface chemistry.^1,10,11^ The surface of the particles can be decorated with various ligands, for example, polymers of different internal structures and chemistry, liquid crystals, proteins, etc.^1,12^ Moreover, surface-patterned particles can be produced. A good example is here the patchy particles that contain domains with different chemical or physical properties. Janus particles with two distinct parts seem to be the most popular.^13^

The behavior of ligand-tethered particles in uniform systems was the subject of numerous studies summarized in several reviews.^1,14,15^ Most of the research focused on the modeling of the morphology of polymer coatings by changing ligand properties, the grafting density, and the interactions of chains with the environment. In this way, one can tune the particle behavior in a wide range. The properties of polymer canopies were widely explored using molecular simulations.^16−26^ In particular, the reorganization of ligands tethered to nanoparticles under different environmental conditions was studied.^22−26^ Dong and Zhou^22^ performed coarse-grained simulations for particles modified with block copolymers or mixed polymer brushes to investigate their responsive behavior in different solvents. Depending on the nature of polymer coatings and solvents, different structures were found: typical core–shell, Janus-type, bucklelike, ringlike, jellyfish-like, and octopus-like morphologies. In some cases, the reconfiguration inside the polymer shell results in the formation of “patchy” nanoparticles.^23,24^ The reconfiguration of the tethered chains can be also caused by the adsorption of isotropic particles on the canopies.^25,26^

Fluid–fluid interfaces involving hairy particles also were extensively investigated.^4−9,27−37^ The surface modification changes the contact angle of particles and their adsorption at the interface. Depending on the nature of attached polymers, the interaction of the particle with a fluid interface can vary from repulsive to attractive. Another important factor is the grafting density; in the dense polymer layers, the chains are stretched while the sparse polymer layers allow a polar liquid to penetrate, causing the brush to collapse. The nature of the grafting bond also plays a considerable role; for example, the thiol–gold bond is relatively mobile, allowing thiolated ligands to move along the nanoparticle surface, while polyelectrolyte brushes grown from the surface of silica particles are irreversibly attached.^38^ A very important feature of hairy particles is that the effective length of grafted ligands is usually comparable to the size of the core. Therefore, the polymer configurations and rearrangements can completely change nanoparticle interactions in the bulk fluids, as well as interparticle interactions at fluid interfaces. In contrast to the particles with a “hard” internal structure, hairy particles change their configurations in response to the surrounding environment. If a particle is adsorbed at a fluid–fluid interface, the chains on the two sides of the interface can adopt different configurations depending on the nature of the ligands, the solvent quality, the grafting density, and other parameters.^38^ The structure of individual hairy particles trapped at the interface was studied by molecular simulations.^27−30,38^ Quan et al.^27^ investigated the structural properties of gold nanoparticles modified with amphiphilic brushes at the oil–water interface. They analyzed the effects of grafting architecture (diblock, mixed, and Janus brush-grafted particles) and hydrophilicity of polymers on the particle morphology and showed that functionalized gold nanoparticles presented abundant morphologies including typical core–shell, Janus-type, jellyfish-like, etc., in different solvent environments. Moreover, it was shown that Janus brush-grafted particles had the highest interfacial stability and activity. Tang et al.^28^ studied hairy particles in a polymer bilayer formed by two mutually immiscible polymers and showed how the grafting density and interactions with the free polymers affect the free energy profiles. They proved that the free energy of rearrangement of the ligands significantly contributes to the total change in free energy upon adsorption of the particle. Tay and Bresme^29^ simulated the alkylthiol passivated gold nanocrystals adsorbed at the air–water interface and demonstrated that the shape of the hairy particles was strongly perturbed by the interface and the length of the ligands affected their wetting behavior. An important role of particle reconfiguration was confirmed by dissipative particle dynamics simulations of Brownian diffusion of Janus nanoparticles at water–oil interfaces.^30^ The diffusion was found to be significantly slower than that of homogeneous nanoparticles. It should be stressed, however, that a good agreement between experimental and simulation results was obtained only when the flexibility of the particle shape had been taken into account. The tethered chains were deformed and oriented at an interface so that the effective radius of Janus nanoparticles was larger than the nominal one obtained by measuring the diffusion in bulk solution.

Self-assembly of hairy particles at the fluid–fluid interface was also intensively studied.^9,31^ Numerous experimental works showed that the hairy particles form both disordered monolayers and different 2D structures of a high degree of ordering.^4−9^ The experimental investigations of the assembly of hairy particles at fluid–fluid interfaces were supported by molecular simulations.^32−34,36,37^ Grest and co-workers carried out atomistic molecular simulations of hairy particles in solutions^16−18^ and at the liquid–vapor interface.^32,33^ They observed a spontaneous asymmetry of spherical hairy particles in solutions and at the liquid–vapor interface. They also showed that varying the terminal groups of a nanoparticle canopy strongly alters the coating shape at the water liquid–vapor interface which leads to different assembly morphologies (short linear clusters with a highly aligned structure, dimers, and disordered clumps).^33^ Gupta and Escobedo^34^ used nonequilibrium molecular dynamics simulations to study the driving forces behind the formation of highly ordered, epitaxially connected superlattices of polyhedral-shaped nanoparticles at fluid–fluid interfaces. Tang et al.^28^ studied the assembly of spherical hairy nanoparticles at the polymer–polymer interface. Depending on assumed parameters, the particles formed a variety of unusual nanostructures, such as dimers with tunable tilt relative to the interface, trimers with tunable bending angle, and anisotropic phases, including serpentine and branched structures, ridged hexagonal monolayers, and square-ordered bilayers. However, in the next work,^35^ they showed that hairy nanocubes can assemble into a variety of unusual architectures, such as rectilinear strings, close-packed sheets, bilayer ribbons, and perforated sheets. Recently, the tailoring interfacial organization of hairy Janus nanoparticles through entropy was studied using dissipative particle dynamics simulations.^36,37^

The behavior of Janus particles at the fluid–fluid interfaces is often described in the framework of the phenomenological model, originally proposed by Pierański^39^ for spherical homogeneous particles and in the next years extended for different anisotropic ones.^9,40−44^ In this approach, the change in free energy of the system associated with trapping a particle into the fluid–fluid interface is expressed by means of surface tensions of all interfaces and the corresponding surface areas. Then, using the Young equation the free energy is written as a function of contact angles of different sides of the particle. This simple model surprisingly well predicts the orientation of Janus particles at the interface and their vertical displacement observed in experiments and molecular simulations.^40−48^ Particle-laden layers were also studied using the density functional theory.^49^

In this work, we use molecular dynamics to study the behavior of hairy Janus particles at a liquid–liquid phase boundary. First, we consider the adsorption of isolated hairy particles and discuss the transformation of their shape on different interfaces. We also try to interpret the simulation data in the framework of a simple phenomenological model. Second, we study the self-assembly of selected hairy Janus particles at the liquid–liquid interface.

The Article is organized as follows. In the next section, we describe the model used and the simulation protocol. Section 3 contains the presentation of the results and their discussion. We start with the analysis of the simulation results obtained for individual hairy Janus particles and compare them with the theoretical phenomenological model. Then, we discuss the self-assembly of these particles. The last section concludes our study.

## 2 Model and Simulation Methodology

We introduce a coarse-grained model of the system that contains two immiscible fluids, a polar fluid W and an apolar fluid O, and Janus hairy particles. A single hairy particle is modeled as a spherical core with attached linear chains of two types. The first kind of ligands consists of segments A while the other comprises segments B. To model a ligand-grafted Janus nanoparticle, the A chains were grafted on one side of the core while the B chains are located on another side. The tethers are randomly distributed over the corresponding hemispheres. We assume that all chains have the same length (each ligand consists of *M* segments) and the numbers of different ligands are identical, *f*_A_ = *f*_B_. To limit the number of parameters, we assume that all segments and molecules of both fluids have the same diameters (σ), while the core diameter is equal to σ_C_.

The chain connectivity is assured by imposing a finitely extensible nonlinear elastic (FENE) segment–segment potential
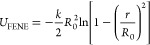
1where *r* is the separation distance between the segments, *k* is the spring constant, and *R*_0_ is the maximum possible length of the spring.

The interactions between all “atoms” (fluid molecules, cores, and segments) are modeled through the shifted-force Lennard-Jones potential^50^
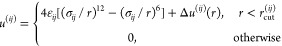
2where
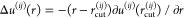
3and *r*_cut_^(*ij*)^ is the cutoff distance, *r*_cut_^(*ij*)^ = 0.5(σ_*i*_ + σ_*j*_) (*i*, *j* = C, A, B, O, W), and ε_*ij*_ denotes a parameter that characterizes interaction strengths between spherical species *i* and *j*. The indices C, A, B, W, and O correspond to the cores, segments A, segments B, a polar fluid W, and an apolar fluid O, respectively. We use the cutoff distance to switch on or switch off attractive interactions. For attractive interactions, *r*_cut_^(*ij*)^ = 2.5σ_(*ij*)_, while for repulsive interactions, *r*_cut_^(*ij*)^ = σ_(*ij*)_. In numerous simulations of hairy particles at fluid–fluid interfaces, the analogous models of particles were used.^24−27,34,35^

The standard units are used. The diameter of segments is the distance unit, σ, while the segment–segment energy parameter, ε, is the energy unit. The mass of a single segment is the mass unity, *m*_s_ = *m*. The basic unit of time is τ = σ(ε/*m*)^1/2^. The gravity effect is assumed to be negligible.

We use standard reduced quantities, reduced distances l* = *l*/σ and reduced energies E* = *E*/ε, and the reduced temperature is T* = *k*_B_*T*/σ, where *k*_B_ is the Boltzmann constant. The standard parameters of the binding potential (eq 1) are introduced, *k* = 30 and *R*_0_ = 1.5σ. The temperature is kept at T* = 1.

We define also the reduced density of the *k*th component as ρ_k_^*^ = ρ_*k*_σ_*k*_^3^, where ρ = *N*_*k*_/*V* is its number density, *N*_*k*_ is a total number of “atoms” *k*, and *V* is the volume of the system.

We carried out simulations for the Janus particles which have similar parameters to homogeneous hairy particles studied by Tang et al.^28^ As in this work, we consider the particles of diameter σ_C_^*^ = 6 and mass *m*_C_^*^ = 216, the relatively short chains with *M* = 10, and the quite high grafting density (the total number of ligands *f* = *f*_A_ + *f*_B_ = 46). Nevertheless, one should remember, however, that in the framework of the FENE model, one segment contains several chemical groups. To limit the number of parameters, we assume that the majority of interactions between species are repulsive. The considered interaction parameters are collected in the next section.

Molecular dynamics simulations were performed using the LAMMPS package.^51,52^ The Nose-Hoover thermostat was used to regulate the temperature. The time step was Δ*t* = 0.005τ. The simulation box was a cuboid of reduced dimensions equal to *L*_*x*_ = *L*_*y*_ = *L* and *L*_*z*_ along the axes *x*, *y*, and *z*, respectively. Standard periodic boundary conditions in the *x* and *y* directions were assumed, and *L* ranged from 70 to 110. In the *z* direction, two repulsive Lennard-Jones (12–6) walls were used to close the system. The size *L*_*z*_ was large enough to obtain two uniform bulk phases: W-rich and O-rich ones.

Each Janus particle is constructed by placing the appropriate number of chains A and B on each hemisphere at randomly selected points. The equator of the core is the Janus boundary.^41^ First, we performed preliminary simulations to check whether the tested particles would adsorb on the interface. A particle was introduced into the bulk fluid, and we observed its behavior. For assumed parameters, the particle gradually moved to the interface (as seen in Figures S1 and S2 in the Supporting Information). Then, we carried the “main” simulations of the particles at the interface. To speed up the calculations, the needed number of Janus particles were introduced to the box and placed at the plane parallel to the *x* and *y* axes at *z* = 0. Next, equal numbers of molecules of the fluids W and O were inserted into the simulation box. We considered particles with different distributions of chains on the core. Examples of initial configurations of individual particles are presented in Supporting Information Figure S3. In the case of systems containing many particles, we started from the configuration shown in Supporting Information Figure S4. During the simulation, Janus particles could freely rotate, translate, and change their conformations.

We equilibrated the system for at least 10^7^ time steps until its total energy reached a constant level, at which it fluctuated around a mean value. The production runs were for at least 10^7^ time steps. At the time, data were saved after every 1000 time steps and used for the evaluation of the needed quantities. To improve accuracy all averages were obtained from several independent simulations.

At the equilibrium, a very well-pronounced phase boundary between these phases is observed. We carried out a series of simulations for a single Janus particle in the system involving the interface. The goal of these simulations was to estimate the position of the adsorbed particle, its orientation, and the shape of the polymer canopy. Next, we performed simulations for many Janus particles trapped at the interface to explore their self-assembly.

We calculated the local densities of both fluids, cores, segments A and B, as well as the observables characterizing the orientation of adsorbed particles and their shapes.

## 3 Results and Discussion

### 3.1 Description of the Studied Systems

Numerous studies showed that the flexible polymer ligands grafted on a nanoparticle can control its properties at interfaces.^9,31,38^ Inspired by these studies, we will show how the strengths of interactions with the fluids affect the deformation of the polymer canopy which, in turn, decides on the behavior of hairy particles at the fluid–fluid interface. In the studied particles, the opposite hemispheres of the cores are modified with ligands that are identical in all aspects except that they are compatible with different fluids.

The behavior of the system depends on the strengths of interactions between all single entities: cores, segments, and fluid molecules. Various combinations of the interaction parameters were considered in simulations of hairy particles in bulk systems.^25,26^

In this work, we assume that self-interactions between fluids are attractive and that cross-interactions are repulsive. For attractive interactions, *r*_cut_^(*ij*)^ = 2.5σ_(*ij*)_ and ε_WW_^*^ = ε_OO_^*^ = 1 while, for repulsive interactions, *r*_cut_^(*ij*)^ = σ_(*ij*)_ and ε_WO_^*^ = 1.^28^ At the considered temperature we obtained a very well-pronounced phase boundary between these fluids. Moreover, we introduce the following assumptions regarding interactions: (i) core–core interactions and interactions of the cores with the remaining “atoms” are purely repulsive, (ii) self-interactions between all “atoms” but the cores are attractive (ε_ii_^*^ = 1), (iii) interactions between segments A and B are repulsive, (iv) interactions of segments A with fluid W are repulsive while the interactions with fluid O can be different, and inversely, interactions of segments B with fluid O are repulsive while the interactions with fluid W can be different. For repulsive interactions, we always set ε_(*ij*)_^*^ = 1. Similar parameters were in the previous simulations of hairy particles.^14,25,26^

Taking into account interactions of the two sides of Janus particles with both fluids, one can divide them into two groups: the particles with (i) “symmetrical” (“supplementary”) and (ii) noncorrelated interactions. In the first case, segments A attract apolar molecules O with the same strength as segments B attract polar molecules W, ε_AO_^*^ = ε_BW_^*^. In other words, the two sides of the particle have identical deviations of apolarity and polarity from neutral wetting. This corresponds to the so-called supplementary wettability condition.^42^ For, the second type of particles ε_AO_^*^ ≠ ε_BW_^*^. In Janus particles, the ligands A and B have different affinities for different solvents. Therefore, the shape of a Janus hairy particle depends on the nature of the bulk phase used. Examples of configurations of selected particles in bulk phases are shown in Figure S5 (Supporting Information).

### 3.2 Individual Janus Hairy Particles

**Figure 1:**
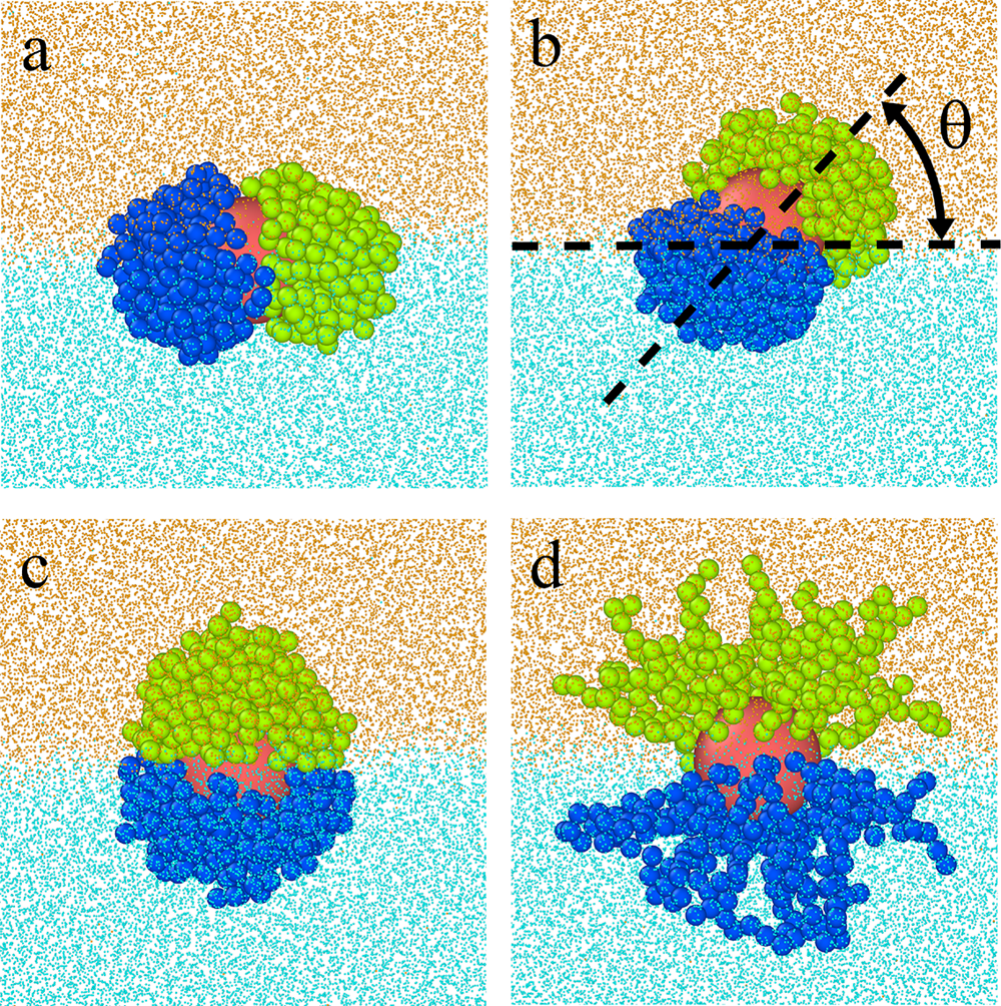
Examples of the equilibrium configurations of hairy Janus nanoparticles with symmetrical AO and BW interactions (ε_AO_^*^ = ε_BW_^*^): (a) repulsive interactions, (b) ε_AO_^*^ = 0.5, (c) ε_AO_^*^ = 1.0, and (d) ε_AO_^*^ = 2.0. The red sphere represents the core; green and blue spheres correspond to A and B segments, respectively. Orange and blue dots represent molecules O and W.

We first examined the behavior of individual Janus hairy particles at the W/O interface. Figure 1 shows exemplary configurations of the particles with “supplementary” interactions with the fluids. We can see that changing the parameter ε_AO_^*^ radically affects the behavior of the particles. The particle shape can considerably deviate from a typical core–shell configuration. When interactions with the solvents are repulsive or weakly attractive (parts a–c), the different ligands collapse at opposite sides of the core and the particle becomes more elongated. The clouds of segments A and B form a kind of “dimer”. On the contrary, if interactions with the fluids are strong, the tethered chains expand to increase the number of more favorable contacts. The chains penetrate the compatible fluids but avoid the interface plane. The resulting particle conformation resembles a sphere “with the indentation in the waist”.

To characterize the orientation of particles, we use the angle between the *xy* plane and the line connecting the mass centers of the clouds of segments A and B (the angle θ shown in Figure 1b). The particles can be adsorbed with the axis parallel to the interface (a), in a tilted (b) or an almost normal (c, d) configuration. The orientation of particles at the interface results from a complex interplay between the reduction of the area of the fluid–fluid interface associated with the adsorption of a particle and interactions between all species. In the case of repulsive AW and BO interactions, the particle lies parallel to the phase boundary to occupy as much area on it as possible. For attractive interactions with the fluids, the behavior of the particle is completely different. As these interactions become stronger, the particle exhibits more apparent Janus properties and gradually changes its orientation, from tilted to normal.

**Figure 2:**
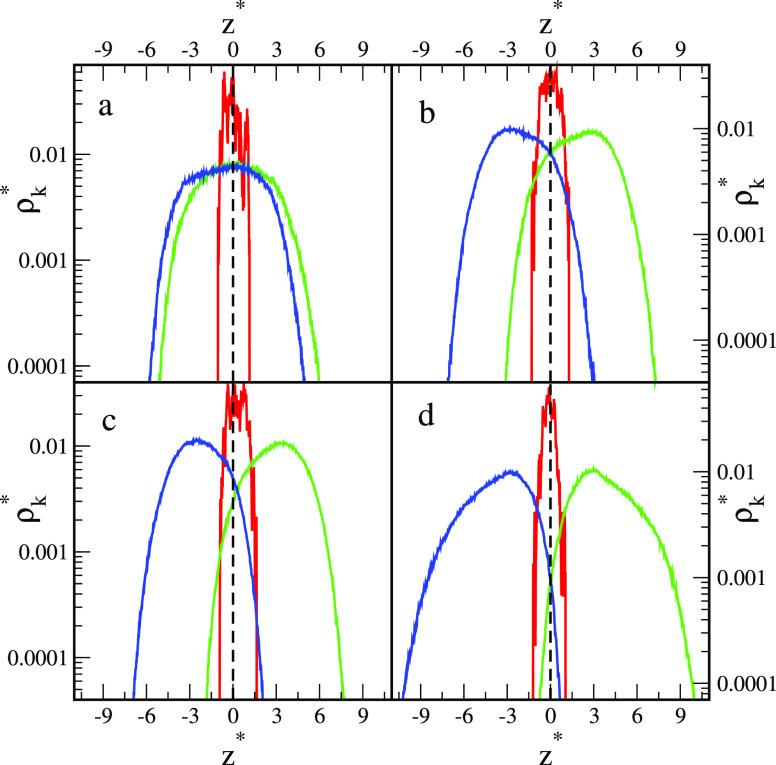
Density profiles of cores (*k* = C, red lines) and the chain segments (*k* = A, green lines; *k* = B, blue lines) of nanoparticles with symmetrical interactions AO and BW interactions (ε_AO_^*^ = ε_BW_^*^): (a) repulsive interactions, (b) ε_AO_^*^ = 0.5, (c) ε_AO_^*^ = 1.0, and (d) ε_AO_^*^ = 2.0. The dashed lines represent the location of the interface. The abscissas are scaled logarithmically.

Figure 2 depicts the reduced core and segment density profiles in the *z* directions for the particles presented in Figure 1. Note that the reduced densities ρ_C_^*^ and ρ_s_^*^ are proportional to the volume occupied by the cores and segments, respectively. All core density profiles have one well-pronounced peak at the phase boundary (z* = 0). As can be expected,^9^ the Janus particles with supplementary interactions are pinned to the boundary no matter how they are oriented and what shape they take. Segment density profiles also have one maximum, but their position and shape depend considerably on the parameter ε_AO_^*^. For repulsive interactions with the fluids, the density profiles are nearly symmetric, have maxima at z* = 0, and decay rapidly with the distance from the interface. The profiles for A and B segments almost coincide. However, a slight shift of the A profile toward the O phase results from the random grafting of ligands to the core. In the case of attractive interactions with fluids, with an increase in the parameter ε_AO_^*^ the segment density profiles shift toward the preferred bulk liquid. However, the A profiles and B profiles still partially overlap. Let us consider the range of z* in which both segments A and B are present. For greater ε_AO_^*^ this “commonly accessible range” gradually decreases. This reflects the fact that the different segments are more strongly separated. In the case of ε_AO_^*^ = 2, the profiles are asymmetrical; the density falls rapidly near the interface, while it decays much slower as the distance from the interface increases.

**Figure 3:**
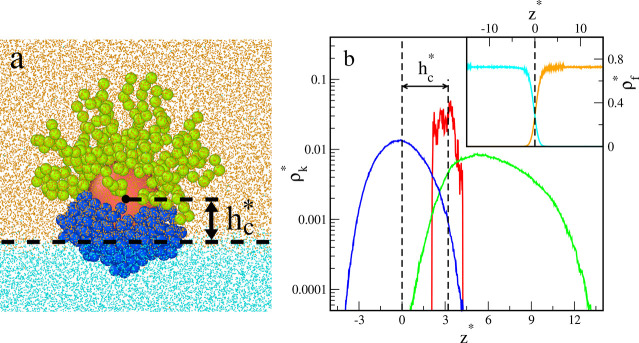
(a) Examples of the equilibrium configurations of hairy Janus nanoparticles for repulsive BW interactions and attractive AO interactions (ε_AO_^*^ = 2.0). The red sphere represents the core; green and blue spheres correspond to segments A and B, respectively. Orange and blue dots represent O and W fluids. (b) Density profiles of the core (*k* = C, red line) and the chain segments (*k* = A, green line; *k* = B, blue line) for the system shown in part (a). The dashed lines represent the location of the interface and the average position of the core. Inset shows density profiles of fluid O (orange line) and fluid W (cyan line). The abscissas are scaled logarithmically.

In Figure 3 the exemplary results obtained for the particle of the other type are shown. In this case, the BW interactions are assumed to be repulsive while the AO interactions are attractive. This causes the particle to be located above the phase boundary with the Janus line almost parallel to it (Figure 3a) The corresponding density profiles are plotted in Figure 3b. The extension of the B chains in the direction of the O-rich phase is clearly visible. To confirm the existence of a well-formed interface, we also present the fluid density profiles (inset).

**Figure 4:**
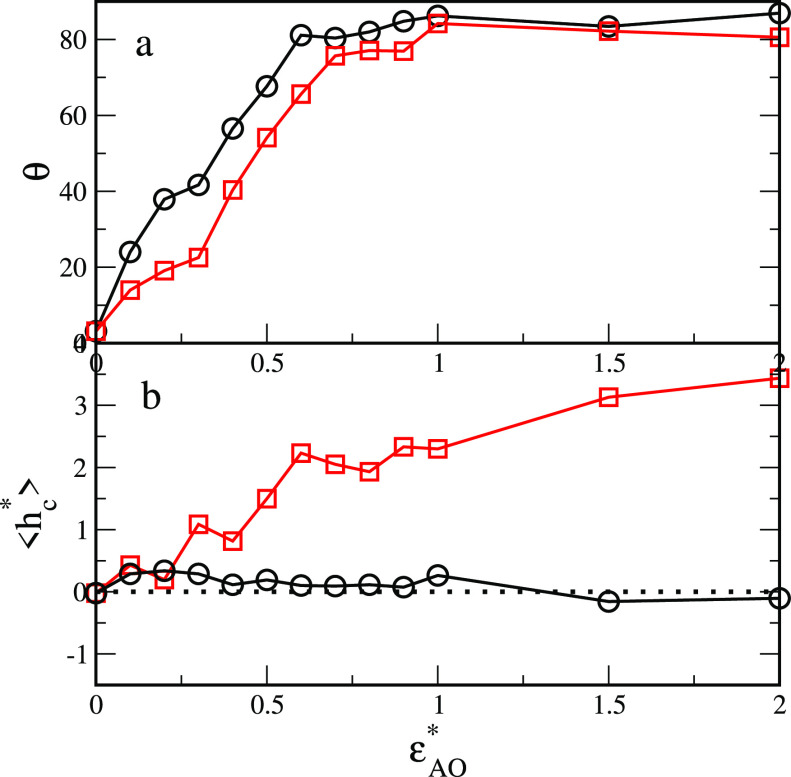
(a) Orientation angle, θ, of the Janus hairy nanoparticle relative to the interface (see Figure 1b) and (b) the average position of the core ⟨*h*_c_^*^⟩ for symmetrical interactions (ε_AO_^*^ = ε_BW_^*^), black lines, and for asymmetrical interactions (repulsive BW interactions and different ε_AO_^*^), red lines. The dotted line represents the location of the interface.

To characterize the configuration of particles at the interface, we estimated the average angles θ and the displacement distances *h*_C_^*^ for both types of Janus particles and the different values of the parameter ε_AO_^*^ (see Figure 4). In the case of supplementary interactions (black symbols), the angle θ quickly increases and for ε_AO_^*^ ≈ 0.6 reaches a plateau, where θ ≈ 90°, while *h*_C_^*^ oscillates around zero. When BW interactions are repulsive and the attractive AO interactions become stronger (red symbols), the angle θ changes similarly as in the previous case. However, for a given value of ε_AO_^*^, the slope is slightly smaller. In this case, the equilibrium displacement distance gradually increases.

Now we turn to analyze the shape of the polymer canopy of the selected particles. We use the shape characteristics defined by means of the gyration tensor^53^
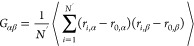
4where *r*_*i*,α_ and *r*_0,α_ are the α component (α, β = *x*, *y*, *z*) of the *i*th segment and the center of mass of the segment cloud, respectively.

Diagonalization of the gyration tensor yields its eigenvalues λ_*i*_ (*i* = 1, 2, 3), which we order as λ_1_ ≥ λ_2_ ≥ λ_3_. Then, three invariants can be obtained *I*_1_ = λ_1_ + λ_2_ + λ_3_, *I*_2_ = λ_1_ λ_2_ + λ_1_ λ_3_ + λ_2_ λ_3_, and *I*_3_ = λ_1_ λ_3_ λ_3_. These values can be used to define the shape descriptors of the segment cloud.^53^

The radius of gyration of the cloud of segments is given as
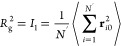
5where **r**_*i*0_ = **r**_*i*_ – **r**_0_, **r**_*i*_ and **r**_0_ are positions of the *i*th segment and the center of mass, respectively, and *N*′ = *fM*.

We resolve the vectors **r**_*i*0_ in eq 5 into components parallel to the axes *x*, *y*, *z* and calculate the corresponding radii of gyration labeled *R*_gα_^2^ (α = *x*, *y*, *z*), the sum of which is equal to *R*_g_^2^. Moreover, we compute the radius of gyration in directions parallel to the fluid–fluid interface, *R*_g*xy*_^2^ = *R*_g*x*_^2^ + *R*_g*y*_^2^.

Then, we can obtain the relative shape anisotropy from the formula
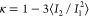
6The relative shape anisotropy takes values between 0 (perfectly spherical objects) and 1 (rigid rods). However, for a regular planar array, κ = 0.25.^53^

**Figure 5:**
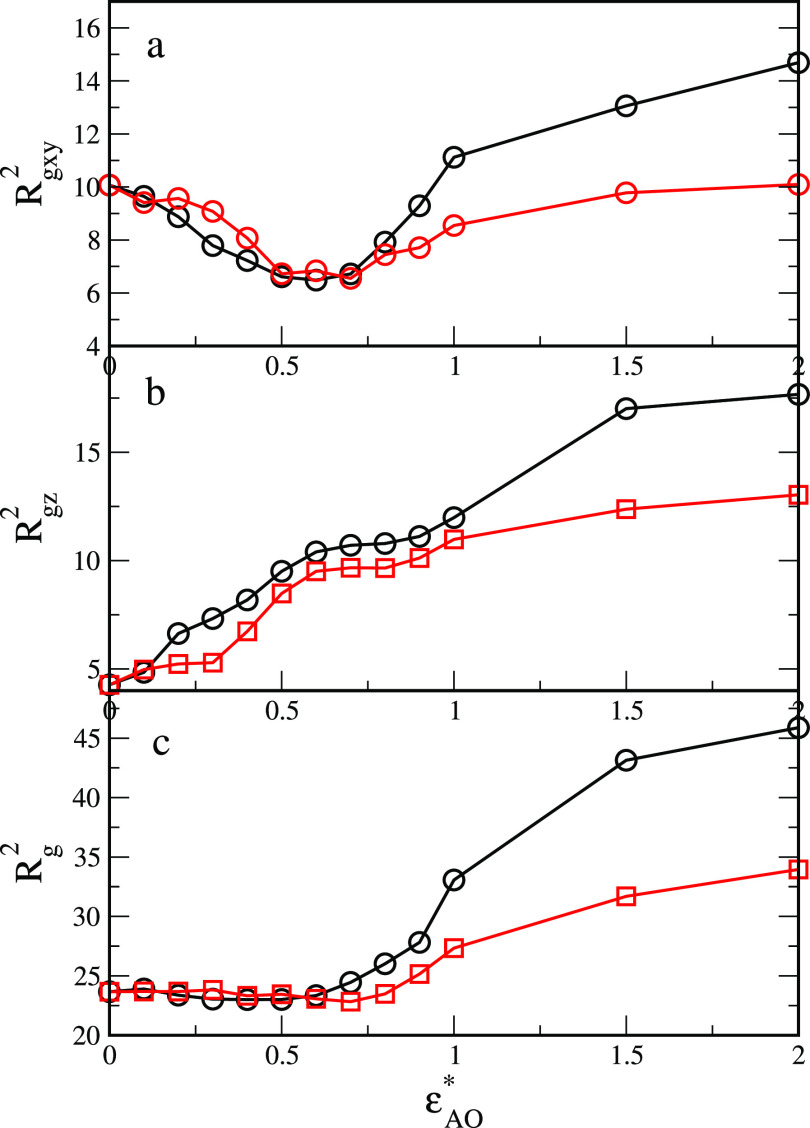
Squared radius of gyration in directions parallel to the fluid–fluid interface (a), the *z*th component of the squared radius of gyration (b), and the total squared radius of gyration (c) as functions of the energy parameter ε_AO_^*^ for symmetrical interactions with the fluids (ε_AO_^*^ = ε_BW_^*^), black lines, and for asymmetrical interactions with the fluids (repulsive BW interactions), red lines.

In Figure 5, we plot the average of the total radius of gyration and its components as functions of the energy parameter ε_AO_^*^.

The squared radius of gyration in directions parallel to the fluid–fluid interface, *R*_g*xy*_^2^ = ⟨*R*_g*x*_^2^ + *R*_g*y*_^2^⟩ , is shown in part a. First, we discuss the particles with the supplementary wetting. For relatively low interactions with the fluids, *R*_*z*_^2^ decreases to the minimum at ε_AO_^*^ ≈ 0.63. This is associated with a change in particle orientation since the particle gets up gradually (see Figure 4 a). However, the chains still remain coiled. A further increase in ε_AO_^*^ causes the polymer layer to swell, which is reflected by an increase in *R*_g*xy*_^2^. In part b, we see that if the parameter ε_AO_^*^ increases the component *R*_g*z*_^2^ monotonically increases. The total radius of gyration (part c) initially does not change because the increase in *R*_g*z*_^2^ is compensated by the decrease in *R*_g*xy*_^2^. For stronger interactions, the effect of chain unwinding dominates, and the particle volume increases. In the case of nonsymmetrical interactions (red lines), the plots are similar to that described above. The significant differences are observed only for high values of ε_AO_^*^ where the radii of gyration are smaller than those for supplementary interactions since the B chains remain coiled.

**Figure 6:**
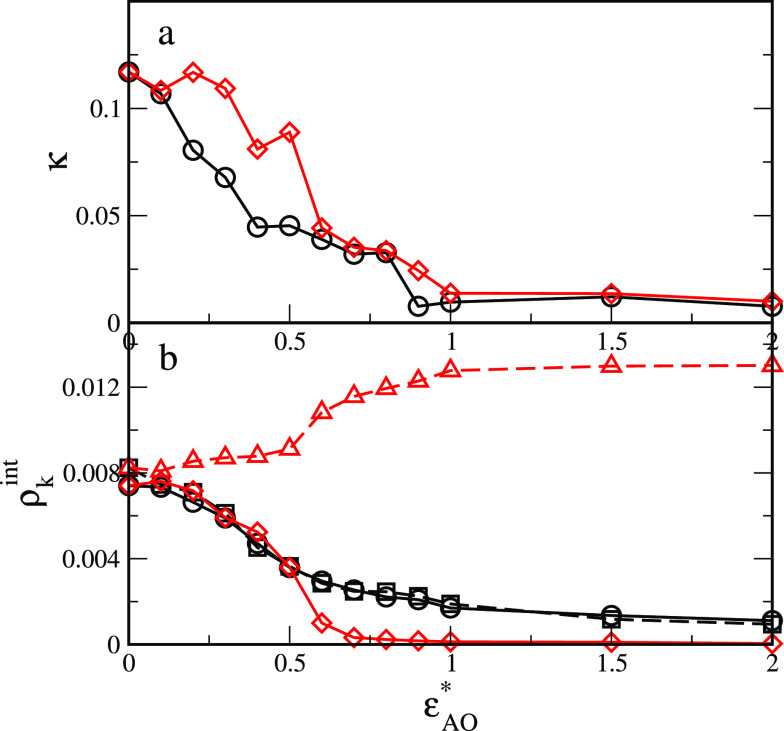
Relative shape anisotropy (a) and densities of segments A and B in the surface layer *z*_C_^*^ ∈ ⟨−0.5, 0.5⟩ (b) plotted for different values of the energy parameter ε_AO_^*^. Black lines and symbols correspond to symmetrical interactions with the fluids (ε_AO_^*^ = ε_BW_^*^), and red lines and symbols are for repulsive BW interactions. Symbols in (b): circles and diamonds, the densities of segments A; squares and triangles, the densities of segments B.

In Figure 6a we show how the shape anisotropy changes with an increase in the strength of AO interactions. In both considered cases, initially, for repulsive interactions, it equals 0.14 and quickly decreases to the value close to the limit value, 0. Thus, when the AO interactions become stronger the particle transforms from a slightly elongated to an almost spherical one.

Moreover, we note that the segment clouds have an indentation near the phase boundary. To quantify this observation, we calculated the densities of segments A and B in the surface layer *z*_C_^*^ ∈ ⟨−0.5, 0.5⟩. In the case of symmetrical interactions, the Janus line is pinned to the interface (Figure 1), and the densities of segments A and B are almost the same (black symbols). The segment densities decrease and achieve a plateau at ε_AO_^*^ = 1. Initially, relatively many segments are near the W/O boundary since the particle lies at the interface. For stronger interactions, the particle gradually changes its orientation, and the chains unfold in the preferred fluids. However, for unsymmetrical interactions (red symbols), the densities of segments A and B in the surface layer are different. In the presented example, the BW interactions are repulsive. For weak attractive AO interactions, the density of segments A changes similarly as previously, but at ε* ≈ 0.75 it falls to zero. This results mainly from a change in both the orientation and the adsorption height. Simply, the segments A leaves the interface layer. Then, an increase of ε_AO_^*^ causes the A chains to stretch. However, the surface density of segments B increases gradually to a certain constant value.

To sum up, our simulations show how the shape of a hairy Janus particle can change during its adsorption at different fluid–fluid interfaces.

### 3.3 Comparison with the Phenomenological Model

To some degree, our particles resemble Janus dumbbells which were intensively studied using different experimental and theoretical methods.^42−46^ The Pierański-type model^9,39^ was commonly used to predict the behavior of these particles at fluid–fluid interfaces. According to this approach, a change in the free energy associated with the transfer of the Janus particle from the phase O to the interface can be written as
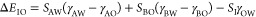
7where γ_*ij*_ denotes the interfacial tension between *i* and *j*, *S*_AW_ and *S*_BO_ are the surface areas exposed to each fluid phase, while *S*_I_ is the area of the O/W interface occupied by the particle when it is attached to the interface. Using the Young equation, the free energy can be expressed by means of the contact angles of different particle sides, γ_OW_cos(θ_*k*_) = γ_*k*O_ – γ_*k*W_, where *k* = A, B. In the same way, we can calculate Δ*E*_WI_ for the transfer from phase W. Unfortunately, the determination of the surface areas of differently wetted parts is difficult, and the analytical expressions were only obtained for a few relatively simple particles. In most cases, different numerical methods must be applied. However, the explicit expression for the adsorption energy can be derived for Janus dimers.^44^

**Figure 7:**
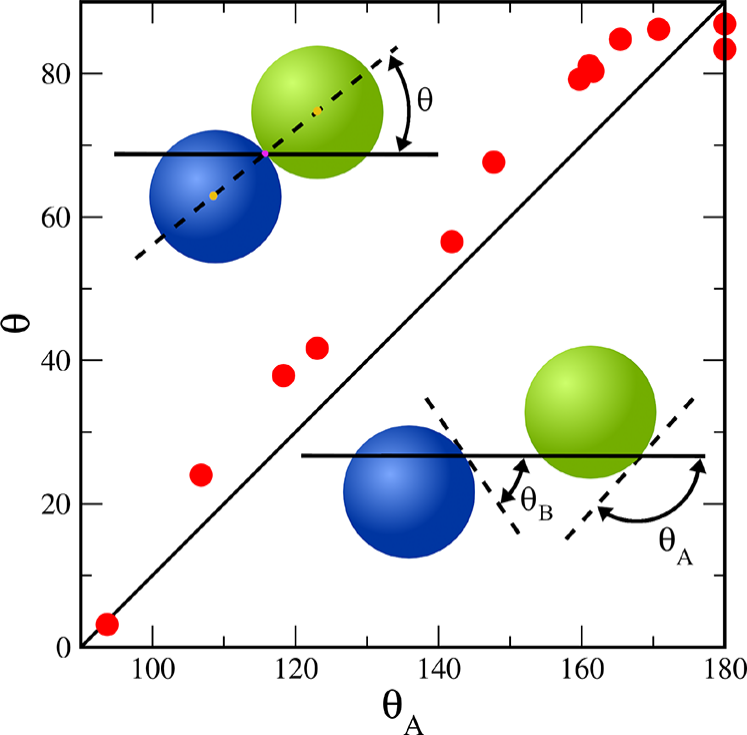
Equilibrium orientation angle of a single Janus hairy particle calculated from eq 10 plotted as a function of the hypothetical contact angle θ_A_. In the top left corner, the orientation angle (θ) of the model dimer is shown. In the lower right corner, the contact angles of the spheres A (θ_A_) and B (θ_B_) are drawn.

Let us consider a dimer build of two spheres A and B. The surface wettability of both segments is represented by the three-phase contact angles, θ_A_ and θ_B_. The contact angle is the angle between the plane tangent to the particle’s surface and the interface at the line where the interface meets the particle (as seen in Figure 7).^42−44^ We assume that θ_B_ ≤ 90° ≤ θ_A_ and θ_A_ + θ_B_ ≤ 180°. In this case, eq 7 has the following form^44^
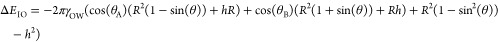
8where *R* is the radius of the spheres, *h* = *h*_C_^*^ is the vertical position of the mass center of the dimer, and θ is the orientation angle of the particle (see Figure 1c and Figure 7).

The equilibrium position and the equilibrium orientation of the Janus dimer can be determined by minimizing Δ*E*_IO_. From the conditions ∂Δ*E*_IO_/∂θ = 0 and ∂Δ*E*_IO_/∂*h* = 0, we have
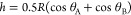
9
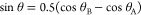
10Note that there is only one minimum; thus, the dimers do not trap in any metastable states.

In the supplementary wettability condition,^9,42^ the spheres of the Janus dimer have the opposite (symmetric) wettability, θ_A_ = 90° + β and θ_B_ = 90° – β, where β = 0.5(θ_A_ + θ_B_). For such systems cos θ_A_ = −cos θ_B_. Putting this relation in eqs 9 and 10, we get *h* = 0 and sin θ = sin β. This means that the particles are pinned at the interface, and the equilibrium orientation angle is θ = β. These dimers adopt a tilted orientation for an arbitrary value of β, but β = 0°. These theoretical predictions were confirmed by molecular dynamics simulation performed for hard Janus dimers.^44^ Moreover, the orientation angle, θ, is a linear function of the contact angle θ_A_, θ = β = θ_A_ – 90°. If θ_A_ = 0 a dimer has the perpendicular orientation, while for θ_A_ = 90° it is adsorbed parallel to the interface.^44^

We propose a toy model for our particles. Each particle is modeled as a dimer built of two spheres A and B. These spheres have the same radius, and we assume that it is equal to half of the radius of gyration of the hairy particle. We have obtained the *h* and θ values obtained from our simulations of hairy particles. Then, from eqs 9 and 10 we calculate the “hypothetical” angles of the parts A and B (cos θ_A_ = arccos(*h*/*R* – sin θ) and cos θ_B_ = arccos(*h*/*R* + sin θ)).

This model can better mimic the shape of particles with symmetrical and rather weak interactions with the fluids (see Figure 1). Therefore, we limit ourselves to the analysis of the results obtained just for symmetrical interactions. In Figure 7 we show the relation between the simulational orientation angles (θ) and the calculated “hypothetical” contact angles (θ_A_), θ = *f*(θ_A_) (points). For each value of θ_A_ we calculated the corresponding “theoretical” angle θ = θ_A_ – 90° (line). The agreement between the theoretical function and simulation data is quite good. Our toy model predicts surprisingly well the orientation of the adsorbed particles. As one can expect, deviations from linearity are greater for stronger interactions (greater θ_A_) since the shape of the hairy particle changes. The phenomenological approach leads to conclusions that are qualitatively compatible with the results of our simulation for Janus hairy particles. This confirms the sense of using simple phenomenological approaches even to model the properties of complex Janus particles.

### 3.4 Self-Assembly of Janus Hairy Particles at a Fluid–Fluid Interface

**Figure 8:**
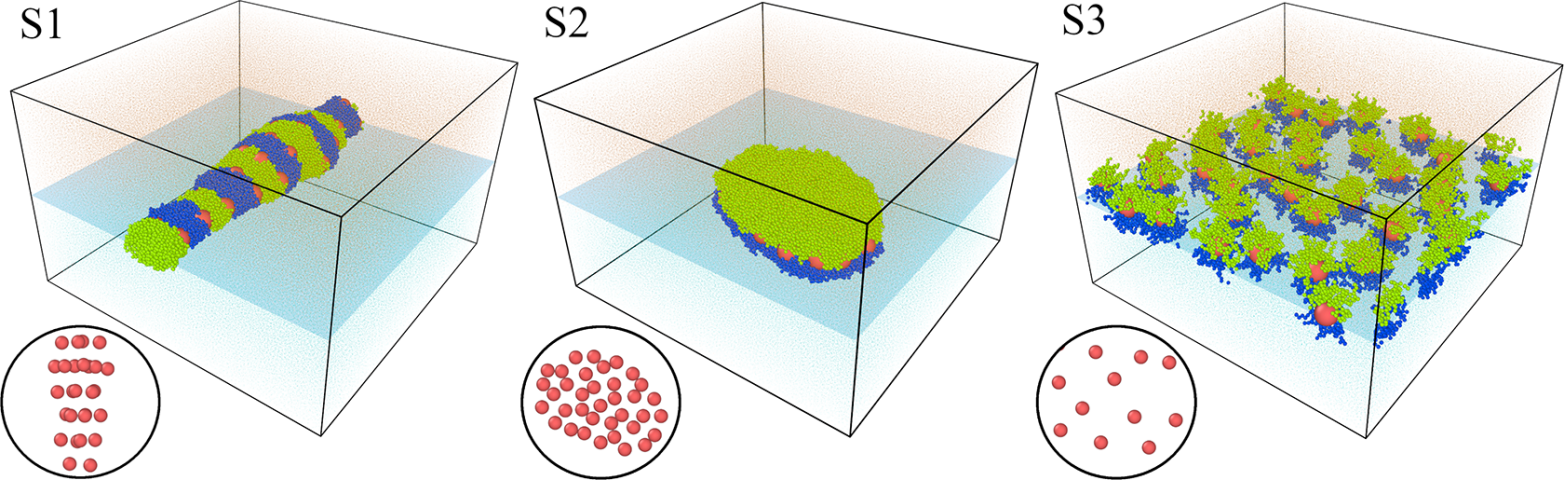
Examples of the equilibrium configurations of different hairy Janus nanoparticles: system S1 (repulsive interactions AO and BW), system S2 (ε_AO_^*^ = ε_BW_^*^ = 0.5), and system S3 (ε_AO_^*^ = ε_BW_^*^ = 1.0). The red spheres represent the cores; green and blue spheres correspond to segments A and B, respectively. Orange and blue dots represent molecules O and W. The blue plane shows the location of the interface. The arrangement of the cores is shown at the bottom (view from above).

**Figure 9:**
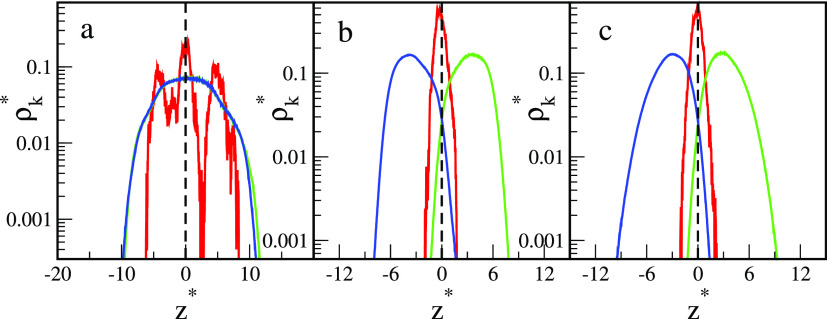
Density profiles of cores (*k* = C, red lines) and chain segments (*k* = A, green lines; *k* = B, blue lines) for the systems shown in Figure 8: (a) system S1, (b) system S2, and (c) system S3. The dashed lines represent the location of the interface. The abscissas are scaled logarithmically.

**Figure 10:**
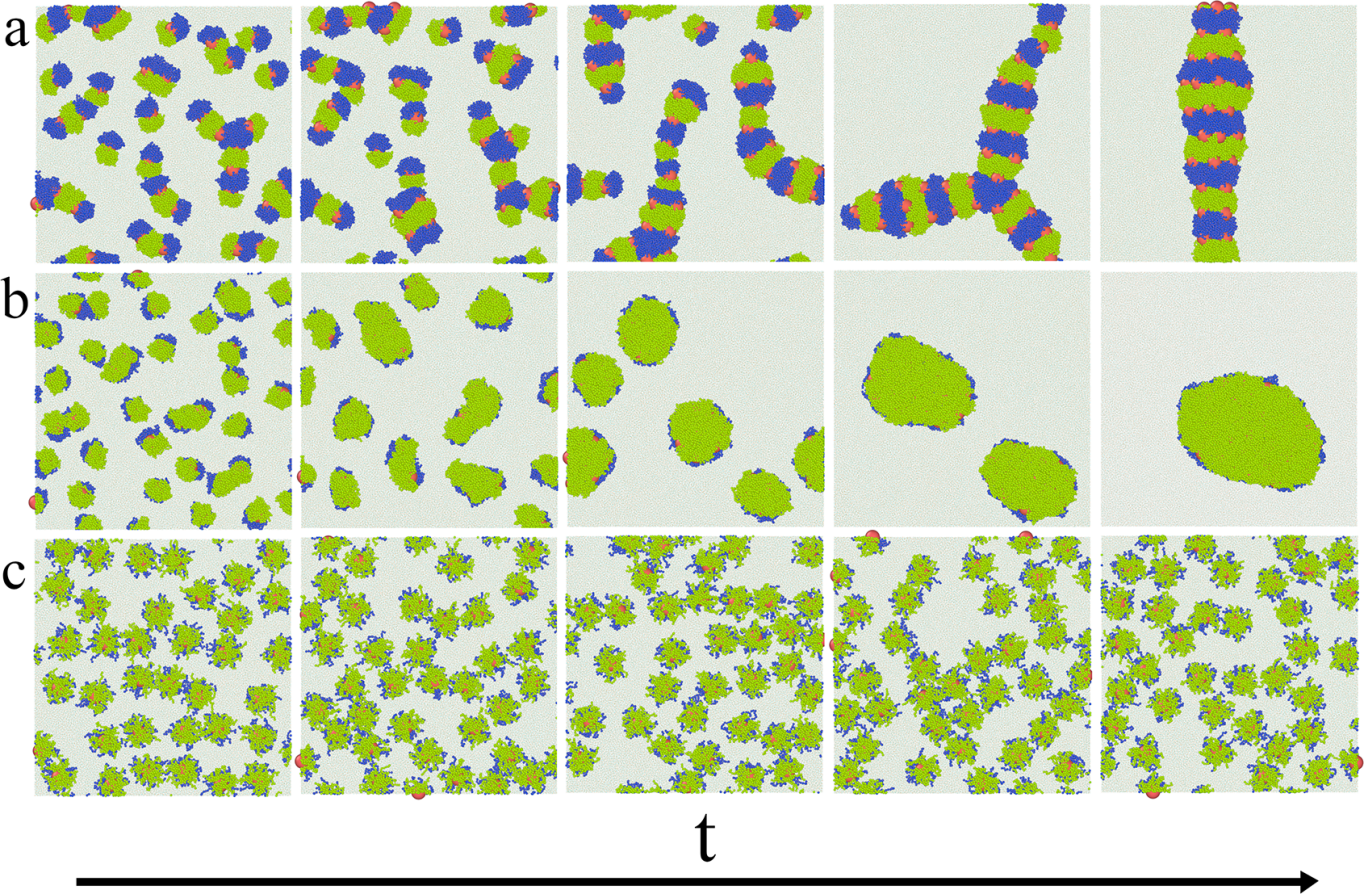
Time evolution of the systems shown in Figures 8 and 9: (a) system S1, (b) system S2, and (c) system S3.

We analyze the assembly in three systems that differ in interactions of ligands with the fluids: system S1 (AO and BW interactions are repulsive), system S2 (ε_AO_^*^ = ε_BW_^*^ = 0.5), and system S3 (ε_AO_^*^ = ε_BW_^*^ = 1). The results are summarized in Figures 8–10, which demonstrate the examples of equilibrium configurations (Figure 8), the corresponding density profiles (Figure 9), and the time evolution of these systems (Figure 10). We study the systems with the surface density Γ* = ∫_–0.5_^0.5^ρ_C_^*^(z*)dz* = 0.7262.

We begin with the discussion of the assembly in system S1 where both sides of Janus particles repel the solvents. In this case, AA contacts, as well as BB contacts, are energetically preferred which in turn promote the aggregation of Janus particles. The resulting giant cluster is presented in the left panel of Figure 8. Let us analyze the internal structure of this aggregate. At the first glance, we see here repeating strips: segments A, cores, and segments B (ACB). However, not all particles lie in the interface plane, but they are also attached to the cluster from the top and from the bottom. This is clearly visible in the density profile of the cores (Figure 9a). There are three “glued together” peaks of cores, which indicate the formation of three layers at the interface. The profiles of segments A and B coincide, so the particles are adsorbed parallel to the interface. It is instructive to discuss the successive stages of the aggregation (Figure 10). Initially, various small clusters are formed, namely dimers (connected via segments B, ACB–BCA, and connected via segments A, BCA–ACB), the strings in which particles are linked through AA, BB, and frequently AB “bonds” and scraps of triple stripes (ACB). The formation of AB bonds requires explanation since the AB interactions are repulsive. However, the interactions between molecules of the same fluid are strongly attractive so they avoid all segments. Thus, solvophobic segments A and B effectively attract each other. Similar, warmlike aggregates of spherical Janus particles were observed experimentally by Park et al.^54^ at the oil/water interface. After some time, small aggregates assemble into long and sometimes branched chains built of stripes ACB. Then, they condense into one cluster. Despite a very long equilibration, the cluster did not transform into a flat aggregate with a characteristic striplike pattern. Likely, the formation of a 3D structure is entropically more profitable.

In the case of system S2, a large oval cluster is formed at the phase boundary. The Janus particles are adsorbed in one monolayer pinned to the interface, with different sides directed toward compatible fluids. The density profiles reflect this observation; the core density has one narrow peak at z* = 0, while the profiles of segments are symmetrically shifted toward preferred fluids. All adsorbed particles have a tilted orientation. It is worth noting that all particles have almost the same inclination angle. In the early stage of aggregation, small clusters of tilted Janus particles are formed. Then, they gradually accumulate into one cluster (Figure 10).

In system S3, however, the Janus particles do not aggregate at all. This somewhat surprising effect can be easily explained. In this case, the AO and BW interactions are identical with the interactions between the same segments. Obviously, attractive interactions between chains belonging to different particles can cause aggregation. It does not happen because the fluid molecules penetrate the brushes and they accumulate near preferred chains. There are many more contacts with fluid molecules than “bonding contacts” between particles. As the result, the adsorption of fluid molecules on chains prevents self-assembly. The density profiles resemble those obtained for isolated particles (compare Figure 9 and Figure 2d). Nevertheless, in very dense systems a typical for repulsive particles hexagonal structure can be formed.

**Figure 11:**
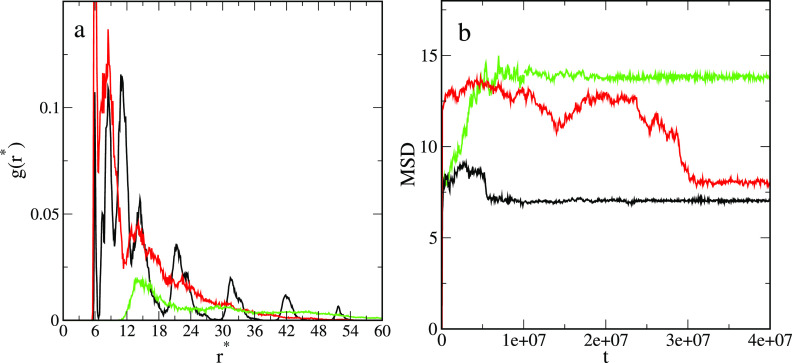
(a) One-particle correlation functions of cores and (b) mean squared displacement of particles calculated for systems S1 (black line), S2 (red line), and S3 (green line) described in Figure 8.

To get a deeper insight into the structure of the aggregates, we analyzed the arrangement of the cores at the interface (see the bottom row in Figure 8). In the case of system S1, the strips of the cores are clearly visible. On the contrary, we do not observe a long-range positional ordering in the remaining systems. In order to confirm our observations, we have calculated the one-particle correlation functions of the cores shown in Figure 11a. For system S1, we see three high maxima (at r* = 6.1, r* = 8.7, and r* = 11.2) and a series of peaks of gradually decreasing heights. The latter reflects a striplike ordering in the system. In the case of system S2, the correlation function has two high maxima at r* = 6.1 and r* = 8.5 and a considerably lower peak at r* = 14.0, but for greater distances, it continuously decreases. In this aggregate, the cores are located closer together than in the previous one. The correlation function calculated for system S3 is completely different. There is one well-pronounced first maximum at r* = 14.1. Thus, the cores are randomly distributed, and the average distances between them are long. In addition for adsorbed states, we computed the mean squared displacement (MSD) of particles to characterize its mobility. Figure 11b shows the mean squared displacement of particles for the systems at hand. We see that the dynamics of particles depends on the assumed interactions. When particles form aggregates (systems S1 and S2), the MSD increases quickly, reaches the first plateau, and then decreases to the second plateau corresponding to the equilibrium state. This reflect the structural evolution of the simulated systems. Initially, particles move quickly at the interface. At this stage, for attractive interactions with the fluids (S2) the particles are more mobile. Then, the particles assemble into small aggregates which later merge into one large cluster. However, when no aggregation takes place (S3), the MSD rapidly increases to the equilibrium plateau.

We consider here only a relatively low surface density of adsorbed particles. Likely, for higher surface densities the ordered monolayers will be formed in systems S2 and S3.^1^ Moreover, various three-dimensional structures can be observed, for example, sandwichlike layers found for hard Janus dimers.^44,49^

**Figure 12:**
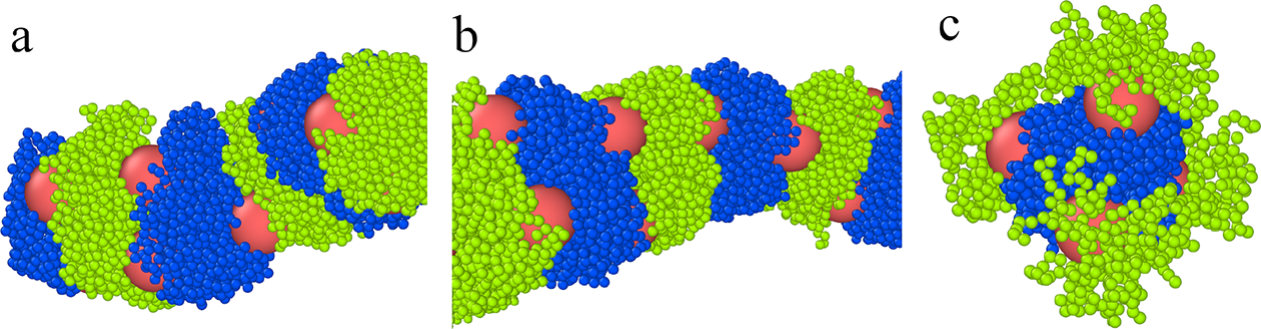
Examples of the aggregates of different hairy Janus nanoparticles in the bulk phase O for repulsive interactions BO and for (a) repulsive interactions AO, (b) attractive interactions AO with ε_AO_^*^ = 0.5, and (c) attractive interactions AO with ε_AO_^*^ = 1.0. The red spheres represent the cores; green and blue spheres correspond to segments A and B, respectively.

We have performed preliminary simulations for the considered particles in the bulk phase O. In all cases, the particles exhibit the tendency to aggregate (see Figure 12). When AO interactions were repulsive or weakly attractive, we found aggregates similar to those observed at the interface in system S1. However, for ε_AO_^*^ = 1 different clusters are formed. This suggests that the presence of the interface can cause the adsorption of unchanged aggregates (S1) or the reconfiguration of clusters (S2) or even can prevent their aggregation (S3).

Our simulations show that by using different fluid–fluid interfaces we can control the assembly of hairy Janus particles.

## 4 Conclusions

We carried out a simulation study of model Janus-like ligand-tethered particles at the interface between partially miscible Lennard-Jones liquids (O/W). Each particle is modeled as a spherical core with chains A and B attached on both halves. The amphiphilicity of Janus particles is controlled by the energies of interactions of the liquids with their polar and apolar parts. To limit the number of parameters, we employed a simple model in which almost all interactions are repulsive. In the study interactions between the same fluid molecules and segments A or B are attractive, Moreover, interactions of segments A with fluid W are repulsive while interactions with fluid O can be different, and inversely, interactions of segments B with fluid O are repulsive while interactions with fluid W can be different. We investigated the behavior of two types of particles: those with symmetrical (supplementary) interactions with the fluids and others with noncorrelated wettability of both parts of Janus particles. Two series of simulations were carried out, for single Janus hairy particles at the liquid–liquid interface and for the systems containing many such particles.

In the first case, we focused on the impact of interactions of particles with fluids on the equilibrium configurations of hairy particles. This configuration is characterized by the vertical position of the adsorbed particle, its orientation, and the shape of the polymer canopy. We have found that the particles with symmetrical interactions are pinned to the interface and have the preferred orientation. However, for asymmetrical interactions, the particle is shifted toward one of the fluids. The shape of adsorbed particles depends on the interactions with the liquids. For weak interactions, the particle is slightly elongated, and two compact polymer layers are formed. To some degree, such a particle resembles a Janus dumbbell. On the contrary, for strong interactions, the chains are unfolded, and the particle seems to be almost spherical. However, there is an indentation in the segment cloud near the interface.

We used the simple phenomenological model of the Janus dimer to predict the vertical position and orientation of the particle. This approach leads to conclusions that are qualitatively compatible with the results of our simulation for Janus hairy particles. We also estimated the hypothetical contact angles of two parts of the Janus particle and used them to “reproduce” the equilibrium orientation of adsorbed particles. The agreement with the simulation data was surprisingly well.

At the second stage of the work, we considered the self-assembly of hairy particles at the liquid–liquid interface. We studied systems with symmetrical interactions. In the case of repulsive interactions with the fluids, Janus particles form various small aggregates which assemble into a giant cluster at the equilibrium. Inside this aggregate, we observed repeating strips: segments A, cores, and segments B. The cluster is a complex 3D structure; although most particles lie on the interfacial plane, some of them are attached to the aggregate from the top and from the bottom. In the case of relatively weak attractive interactions with the fluids, we found a large oval cluster built of Janus particles pinned to the interface, with different sides directed toward compatible fluids. All particles are identically oriented relative to the interface. For very strong interactions with the fluids, Janus particles do not aggregate at the studied surface density. The penetration of the polymer canopy by the fluids prevents self-assembly.

This work shows that the interactions of Janus hairy particles with the fluids forming the interface decide their surface activity and the structure of the particle-laden layers. Our simple coarse-grained model captures the mechanism of adsorption of Janus hairy particles at a fluid–fluid interface, including the shape transformation of the polymer canopies. The simulation results presented here are consistent with experimental studies.^1,9^ Nevertheless, the adsorption and assembly of Janus hairy particles at interfaces require further research associated with the impact on these phenomena by such parameters as the core shape, the nature of ligands, the length of tethers, the grafting density, etc. The role of interfacial deformation on particle orientation and assembly could also be examined in more depth.

Our study extends our understanding of modeling nanoparticles with desired surface activity and controlling the structure of particle-loaded layers. The ability to manipulate the morphology of the interface may generate new opportunities for nanotechnology.

## Supporting Information Available

Figures on simulation details and bulk fluids (PDF)
