# Supplementary material for: Janus Ligand-Tethered Nanoparticles at Liquid–Liquid Interfaces
Source: J Phys Chem B. 2023 May 29;127(22):5150–61. doi: 10.1021/acs.jpcb.3c01943 (PMC10258801; doi:10.1021/acs.jpcb.3c01943)
Supplement: Supplementary file 1 — jp3c01943_si_001.pdf [file jp3c01943_si_001.pdf]

Supporting Information

Janus Ligand-Tethered Nanoparticles at Liquid-Liquid Interfaces

M. Borówko, T. Staszewski*, J. Tomasik

Department of Theoretical Chemistry, Institute of Chemical Sciences,
Faculty of Chemistry, Maria Curie-Skłodowska University in Lublin, Poland

1. Simulation details

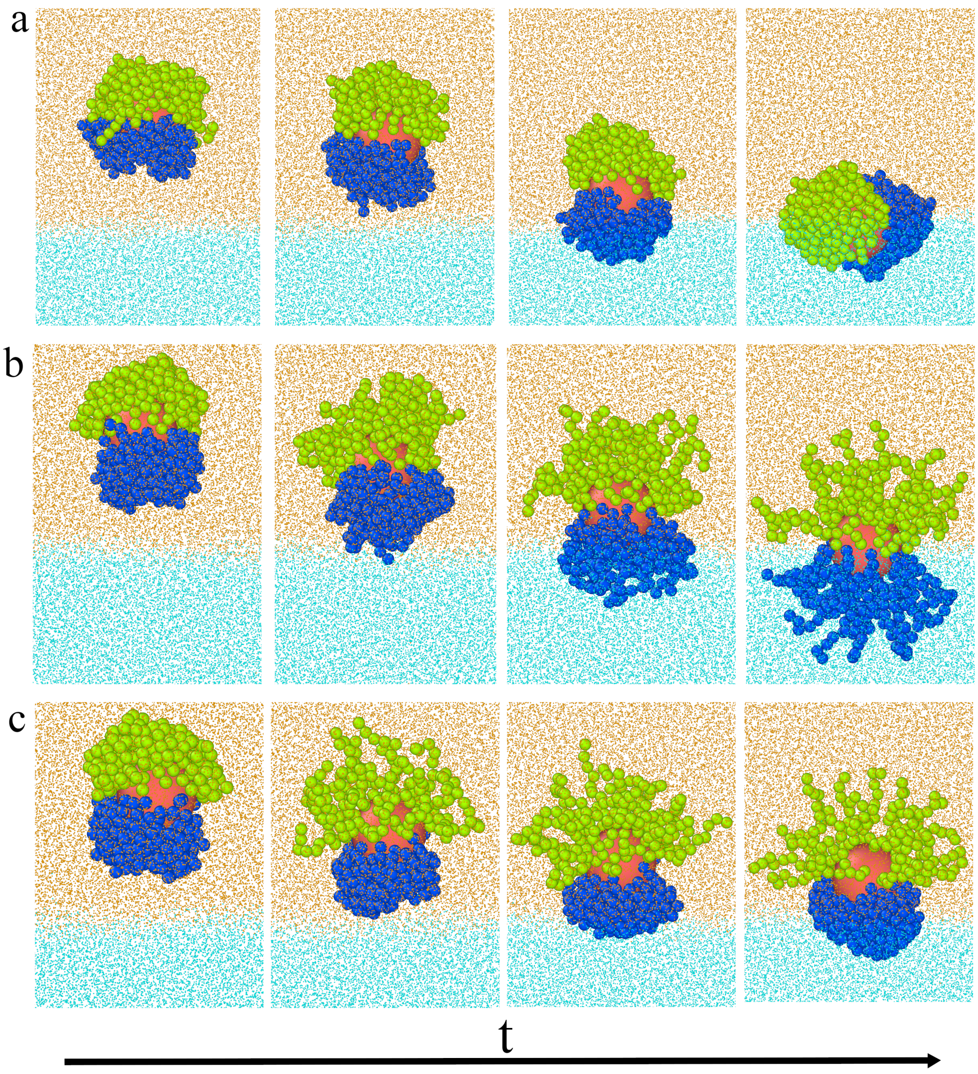

Figure S1. Time evolution of the system containing a particle passing from the bulk phase O to the interface for (a) repulsive AO and BW interactions, (b) attractive AO and BW interactions $\varepsilon^*_{AO}=\varepsilon^*_{BW}=2$, and (c) repulsive BW interactions and attractive AO interactions $\varepsilon^*_{AO}=2$.

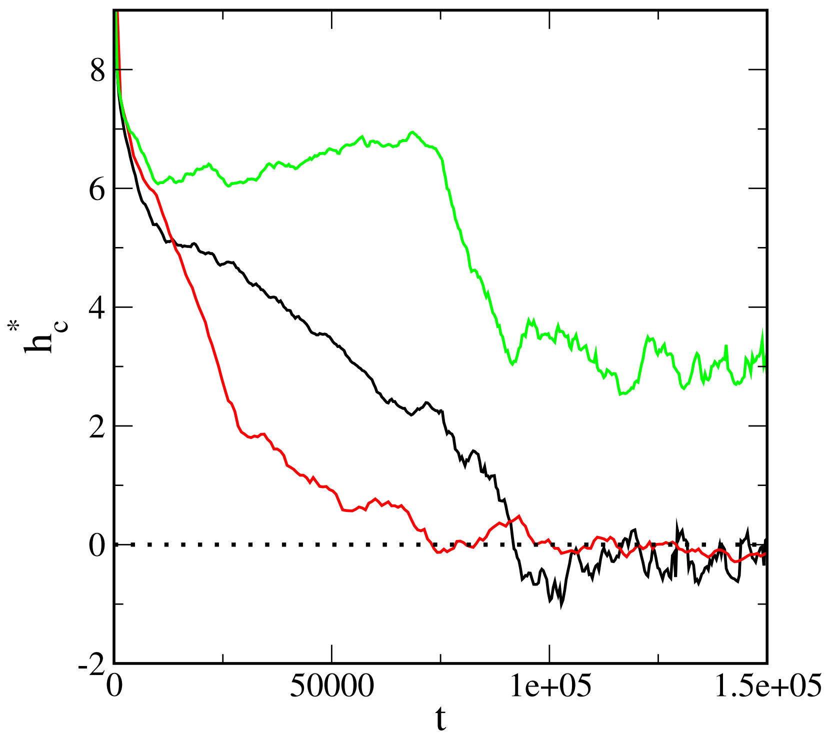

Figure S2. Changes of the distance of the core center from the interface during simulations for the systems from Fig. S1, black line is for repulsive AO and BW interactions, red line is for attractive AO and BW interactions $\varepsilon^*_{AO}=\varepsilon^*_{BW}=2$, and green line is for repulsive BW interactions and attractive AO interactions $\varepsilon^*_{AO}=2$.

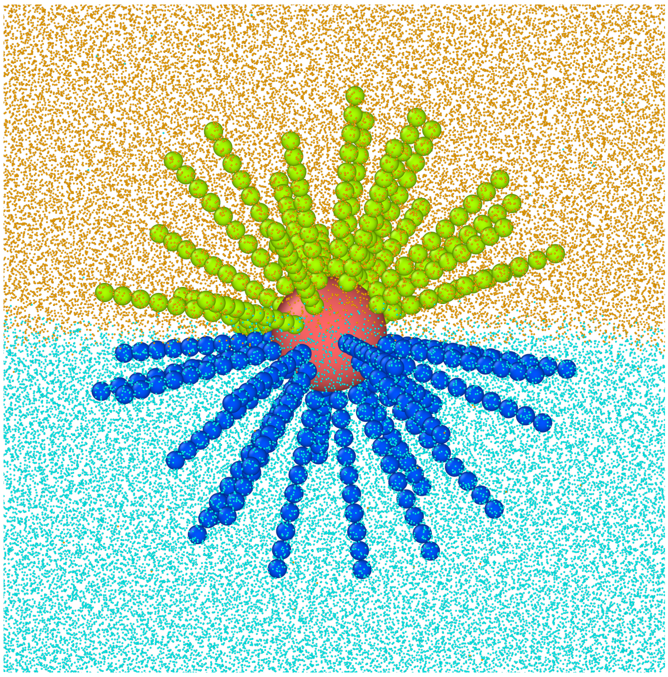

Figure S3. An example of initial configuration for simulation of the behavior of individual hairy particles at the interface.

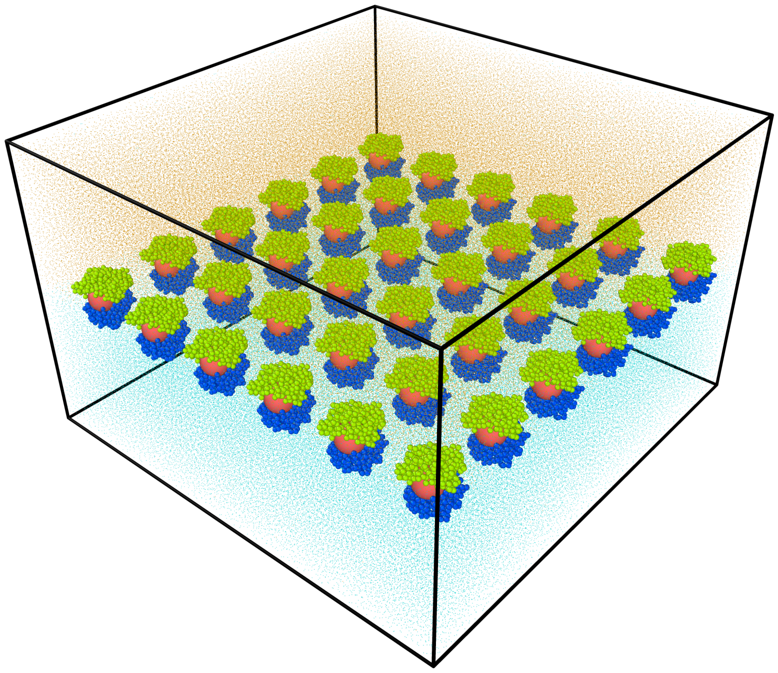

Figure S4. An initial configuration for simulation of the assembly of hairy particles at the interface.

2. Bulk fluids

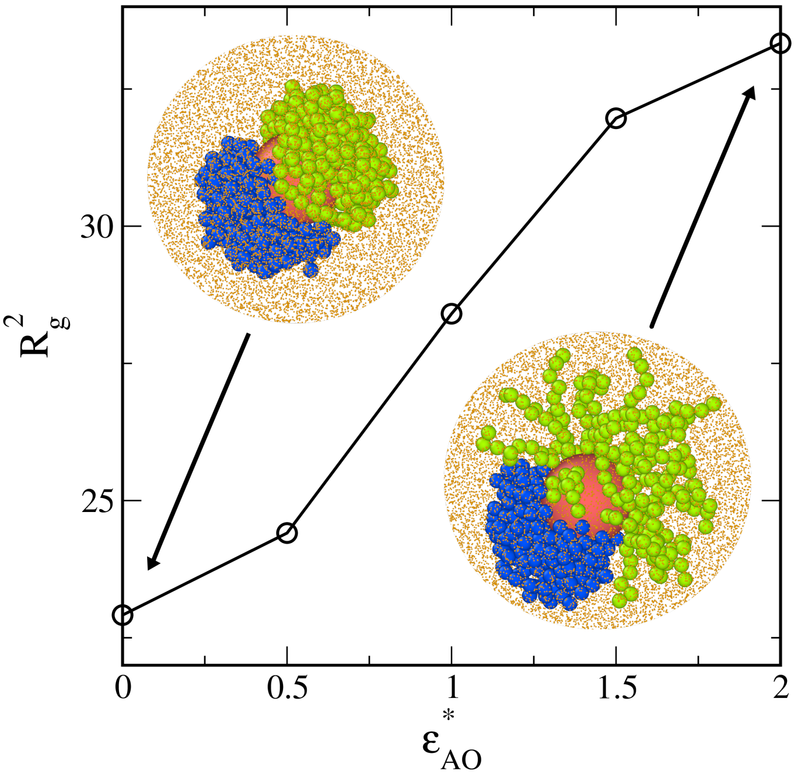

Figure S5. The squared radius of gyration of a hairy particle immersed in a bulk fluid O as functions of the energy parameter ε^*_{AO} for repulsive BO interactions with examples of the particle configurations.
